# Leadership for child health in the developing countries of the Western Pacific

**Published:** 2011-06

**Authors:** Rami Subhi, Trevor Duke

**Affiliations:** 1 Centre for International Child Health, Department of Paediatrics, University of Melbourne, MCRI, Royal Children’s Hospital, Melbourne, Australia; 2 Discipline of Child Health, School of Medicine & Health Sciences, University of Papua New Guinea, Papua New Guinea

## Abstract

The content and landscape of global child health is increasingly complex. There is strong evidence for the effectiveness of local, national and institutional leadership in reducing child mortality, but this has not been a focus of global health initiatives. Interventions to strengthen health systems should include support for local leadership: building-up institutions of training, empowering national paediatric professional associations, creating opportunities for contribution and leadership at national, provincial and local level, and networks of support for staff working in child health in remote areas. In the poorer high mortality burden countries of the Pacific, to meet the clinical and public health gaps, there is a need for increases in the education of child health nurse practitioners, and development of systems of continuing professional development for paediatric doctors and nurses. Involvement in local research, especially that which contributes directly to critical issues in child health policy or strengthening national data systems builds capacity for leadership.

The 524 000 child deaths each year in countries in the Western Pacific are much higher than what would be expected given the body of available knowledge and interventions (1,2). This gap has been attributed to the failure to deliver essential health services in a timely, sustainable and equitable way to children who need them (3). Health systems in the poorer developing countries with high death rates for children are often described as weak. Accounts point to deficiencies in each building block of the system: in the quality of health services, the workforce, health information systems, access to essential medical devices, financing, leadership and governance (4). The need for health systems strengthening is frequently cited (5). Yet, it is only recently that there has been a common definition (3), and that the major players in global health have proposed a joint approach to collaboration on health systems strengthening (6). As yet, the evidence-base for the impact of these approaches on health outcomes is mixed and generally weak (7).

The challenges in achieving a concerted effort to strengthen weak health systems are inherent in the breadth of the WHO’s definition of health system as “all organizations, people and actions whose primary intent is to promote, restore or maintain health” (3). With the multiplicity of partner agencies, their vertical approaches yet calls for integration and harmonization of health and non-health initiatives, the increasing complexity of health care, and many threats from outside and within, countries are challenged in coordinating, setting and sustaining national priorities, and developing quality health services.

In 1997, the Partnership for Maternal, Newborn and Child Health released a statement calling for leadership at global and national levels to achieve the Millennium Development Goals (MDGs) for child (MDG 4) and maternal health (MDG 5) (8). The statement asserted the important role of health professional associations, and called for actions to develop local leadership in high mortality burden countries. In 2005, the Western Pacific Regional Office of WHO launched the Child Survival Strategy. This also included a call for leadership from political figures and policy-makers (9): “Policy-makers in different government sectors must provide strong and consistent leadership. Accelerating child survival efforts will require leadership from influential political figures at the highest possible level. To ensure wide support and that children’s rights to health and health care are addressed, highly visible and well-respected champions will be needed across different sectors of society.”

So what does leadership actually entail for child health? We set out to explore its role in the high mortality burden countries of East Asia and the Pacific: who does or can provide leadership, how to strengthen it, and whether this would have an impact on child health. We argue that this is an unmet need. We explain the reasons why leadership is so important and that in addition to political and policy leadership, the scope of leadership must be extended to clinical and public health leadership at all levels of the health service if child health is to improve.

## IDENTIFYING BACKGROUND EVIDENCE

To discuss these issues with a sound evidence-base we searched PubMed (1980–2011), CINAHL (1970–2011) and Embase (1980–2011) using combinations of the terms ‘leadership’, ‘child’, ‘health’, ‘mortality’, and ‘professional associations/societies’. The search was limited to articles in English. A sensitive search was performed on PubMed, and complemented with more specific searches by omitting some of the keywords on subsequent searches (details available on request from authors). As it was possible that more information on this was available in the grey literature, key terms were also entered in Google Scholar, the New York Academy of Medicine library and the World Health Organization databases. The International Pediatric Association (IPA) database was used to identify professional associations in countries in the Pacific and East Asia. We anticipated a scarcity of literature dealing with these issues. Therefore, we broadened the search to include literature on the impact of leadership on health (not specifically child health) that was relevant to developing and least developed countries. The United Nations Statistical Division classifications were used when referring to ‘least developed’ and ‘small island developing’ countries (10).

There is limited evidence in this field. We identified 16 publications of varying relevance. The search results are summarised here primarily to highlight the paucity of published literature, and the varying interpretations of what leadership for child health means. Five articles dealt with national leadership: three articles from Papua New Guinea (PNG) (11), Mexico (12) and Thailand (13) were specific for child heath, and two articles from Thailand (14) and Sub-Saharan Africa (15) were more general. Three articles discussed the role of professional associations for maternal and child health (16-18), and one outlined the development of paediatric services in Singapore (19). Three articles addressed clinical leadership: two from developed countries evaluated the impact of nursing leadership on patient outcomes (20,21), and one described the postgraduate paediatric training program in PNG (22). Four articles outlined interventions to improve public health and clinical leadership: South Africa’s experience with mortality audit and policy for maternal and child health (23); the development of leadership training for paediatric residents (24) and designing nurse leadership training (25) in the United States; and an evaluation of the Fiji nurse practitioner training program (26). Nine additional reports that were directly relevant were retrieved from the gray literature search (3,4,7-9,27-30). The examples and data retrieved were used as background in the broader discussion that follows.

Data on the health situation for children in the East Asia and Western Pacific Region were sourced from UNICEF, WHO, the published literature, and are cited where used.

## COUNTRIES OF THE EAST ASIA AND WESTERN PACIFIC REGION

The countries of the East Asia and Western Pacific Region are heterogeneous. Based on their child health characteristics, many child health challenges are shared by countries classified by WHO and UNICEF as Group 1 countries (Cambodia, Kiribati, Marshall Islands, Lao PDR, Papua New Guinea, Solomon Islands and Vanuatu), the other Pacific Island nations and Timor-Leste (31,32). In these countries infectious diseases and under-nutrition remain prominent throughout most of the population. While some countries are on-track to achieving MDG 4 targets, no country in the Region is on track for achieving all its Millennium Development Goals. It is for these countries that this review is predominantly relevant. In these countries poverty rates are high; one in four households is below national poverty lines and protein energy and micronutrient malnutrition is common. Other shared features include geographical isolation, poor health infrastructure, limited human resources, and in many countries, frequent natural disasters, risks from climate change, limited access to safe water and sanitation, and limited domestic markets. PNG has the highest burden of child deaths in the Pacific region, with about 14 000 child deaths per year. Lao PDR, Timor Leste and Cambodia have among the highest child mortality rates in the East Asian Region. In Pacific Island states more than 70% of child deaths occur in infancy, and neonatal mortality rates have not appreciably fallen in the last 10 years.

## THE NEED FOR LEADERSHIP

### Child health is becoming more complex

There are many reasons why leadership is needed now more than ever. The content and landscape of child health in developing countries has become increasingly complex in the last two decades. Twenty years ago there was no artemisinin-based treatment for malaria, rapid diagnostic tests, antiretroviral therapy, programs for prevention of mother to child transmission, fixed-dose combination therapy for tuberculosis, insecticide-treated bed nets, Integrated Management of Childhood Illness, zinc or vitamin A, conjugate vaccines against *Pneumococcus* or *Haemophilus influenzae* type b, rotavirus or Human Papilloma Virus, little use of third-generation cephalosporins or concerns over antibiotic resistance for common infections such as meningitis and dysentery. Twenty years ago there was little focus in developing countries on the care of the neonate, the needs of adolescents, child welfare, disability or human rights. Now all these areas are part of a comprehensive child health program, and to be properly implemented, especially in large or decentralised health systems there needs to be technical and leadership capacity at a national, provincial and a district level. Not only is the content of child health more complex, countries now interact with a global health landscape that has many more players with varied agendas and methods, and varied recognition of local capacity and its importance.

### National leadership, and effective professional bodies can lead to improved health outcomes for children, and strengthen local capacity

While not proving the case for leadership, improvements in child survival in a number of countries in Asia, including Malaysia, Thailand and Singapore, occurred in conjunction with the development of national leadership and strong roles by professional associations for the introduction of national strategies (13,19,27).

In Thailand improved health service quality and equity has resulted from a range of financial and public health reforms. Some of these have been initiated by governments; others were a product of advocacy from professional bodies, including the Rural Doctors’ Society (13). While not specific for child health, this Society, founded in 1978, initiated management training programmes, developed management handbooks and designed activities to support rural district hospital doctors. These included publication of a rural doctor journals/newsletters, public recognition for extraordinary performance, coordinating visits to rural hospitals by senior doctors to improve morale and organising provincial rural doctor coaches. The Rural Doctors’ Society supported essential medicines policy, highlighted corruption, and advocated for better conditions for rural health professionals (14).

In Singapore, the first group of child health leaders initiated the national program of Maternal and Child Health Welfare services. The program included promotion of breastfeeding, and healthy birthing and rearing practices, and led the paediatric training in medical schools, training the country’s first paediatrician in 1932. Associated with these was a reduction in infant mortality in rural areas in Singapore from 263 per 1000 live births in 1927 to 86 per 1000 in 1938 (19).

There are examples of national leadership in implementing vertical programs. Malawi’s recent success in scaling-up anti-retroviral therapy – a product of strong national leadership and coordinated effort by partner agencies – has been associated with a reduction in adult mortality rates, and a significant increase in the number of HIV-infected adults on long-term anti-retroviral therapy (15).

Strong links have been drawn between the work of professional societies of obstetrics and midwifery and reductions in maternal deaths in many countries in a wide-ranging review (16). Historical and current links are described, as are the roles of such professional organizations. In 19th Century Sweden – one of the first countries to adopt national policies for maternal health and promote professional midwifery practice – the rate of maternal mortality was half of the rest of Europe at a time before antibiotics and anaesthetics. There are also published experiences of the positive impact that the collaboration between the Association of Obstetrics and Gynaecologists in Guatemala and the Society of Obstetricians and Gynaecologists of Canada has had on building institutional and individual leadership capacity for women’s health in Guatemala (17).

## NATIONAL LEADERSHIP, AND THE ROLE OF PROFESSIONAL BODIES IN THE WESTERN PACIFIC REGION

The Western Pacific Regional Child Survival Strategy called each country to have a single national child health plan, and mechanisms for coordinated implementation of programs, monitoring and evaluation (9). Some countries in the Western Pacific Region have adopted this Strategy, but few of these are Pacific Island States (33). Where progress has been achieved, such as in PNG, the processes is generally led by a central coordinating committee, which is composed of representatives from the ministry of health and a permanent national representative body (professional association or society) for child and maternal health, university academics in child health, UN agency representatives and community groups (11). Such local institutional organisations do not exist in many countries that have not taken up the strategy.

National paediatric societies, working closely with ministries of health, can be effective in upholding the child health agenda by accepting responsibilities for providing technical advice on national child health priorities, maintaining standards for clinical care, leading child health training, and advocating and lobbying for broad child health issues and turning policy into practice (**Table 1**).

**Table 1:** The roles of a national paediatric association or society

To develop and maintain standards of paediatric clinical care and public health, according to the latest evidence. The Paediatric Society is the custodian of these treatment policies, and ensures they are kept up to date.
To provide technical advice to the Ministry of Health on all aspects of child health, including social, environmental, developmental, curative and preventative health. The advice should be based on evidence and professional experience and wisdom.
To provide advice to the community on important aspects of child health, for example through public awareness campaigns about breast-feeding, immunization, nutrition and school attendance
To link with institutions of training to provide input on health training curricula, so that nursing, under-graduate medical and other health worker courses reflect the national child health policies and guidelines
To develop continuing professional development for paediatricians and child health nurses to ensure the maintenance of professional skills, knowledge and standards
To be a collegiate society providing professional and personal peer support

## PROFESSIONAL ASSOCIATIONS FOR CHILD HEALTH IN THE PACIFIC

**Figure 1:**
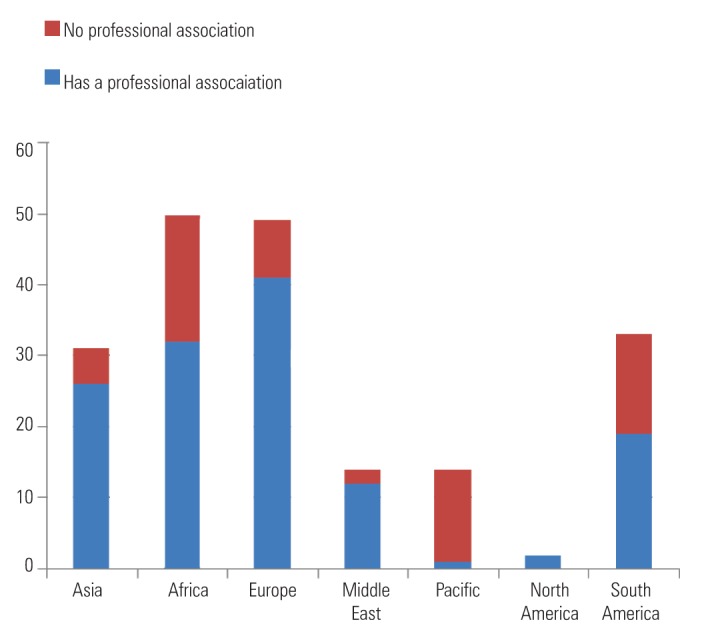
Number of countries in each region with professional associations for paediatrics (34).

Of 194 countries throughout the world, 132 have professional associations registered with the International Pediatric Association (IPA) (34). There is regional variation in the proportion of countries with paediatric associations (**Figure 1**). For 13 of the 14 Pacific Island countries, and for Timor-Leste, there is no registered association. PNG is the exception in the Region, where the Paediatric Society has led child health policy, the development of paediatric training, local treatment guidelines and programmes for equipping generalists in managing children in rural areas, public health initiatives such as the introduction of new vaccines, and the integration of global strategies (11).

Lack of registration with the IPA does not mean there are no informal national leadership groups for child health. For example, Fiji and the Solomon Islands have committees overseeing paediatrics and child health, with the potential to fulfil many of the same roles that a registered paediatric society can. However in general, without a formal paediatric association, the capacity for fulfilling national leadership roles is thinner.

## LOCAL AND INDIVIDUAL LEADERSHIP

With the decentralisation of health systems in the Region, provincial or district governments are taking on greater responsibilities (35). The need for technical capacity and leadership in child health programs applies for managers coordinating programs at the district health office, and for health workers at the front-line. Surveys of districts in developing countries have shown deficiencies, amongst others, in program management and evaluation (36) and quality of care (37). Committed and well trained individuals are needed to: oversee child public health programs; manage, understand and use child health data effectively; coordinate the up-skilling of health workers in current treatment approaches for children; advocate and promote standards for good quality care, and communicate with the national leadership for child health. Paediatricians and child health nurses can carry out these roles. However, many such individuals are under-supported, receiving little continuing professional development and no extra remuneration or time allowance.

Many countries will not have a trained paediatrician at every province or district in the foreseeable future. In these countries there is often no cadre of adequately skilled health workers to fill this gap. Pacific Island countries with the highest child mortality rates have low physician densities (4), and therefore nurses are the first-line cadre of health workers caring for sick children. Nurses need advanced training in child health to function effectively, and often, independently. PNG is currently the only country in the Pacific with a program for training child health nurses, and that country now has one program, where there were formerly four. Smaller countries in the Pacific, with even more limited number of practicing paediatricians, have no such training programs. This is in contrast to midwifery training, which exists in all but 6 of the smallest Pacific countries (**Table 2**).

**Table 2:** Child health and midwifery training programs in the Pacific Island countries (29,44)

Country	Under-5 mortality rate	Paediatric nursing training	Midwifery training	Nurse practitioner training
Cook Islands	18–26	No	UNFPA certificate	9 month in-country course for graduate nurses, funded by NZAID
Federated State of Micronesia	40–47	No	Diploma: Fiji School of Nursing & University of South Pacific	No
Fiji	18–22	No	Diploma: Fiji School of Nursing; some nurses training in Tonga and Western Samoa	Fiji Nurse Practitioner Program
Kiribati	63–69	No	Diploma: Kiribati Midwifery Program.	No
Nauru	30–38	No	Diploma: Fiji School of Nursing	No
Niue	19	No	Diploma: Fiji School of Nursing	No
Palau	10–39	No	Diploma: Fiji School of Nursing	No
Papua New Guinea	74	Diploma of child health	Bachelor in University of PNG; diplomas in Pacific Adventist University, University of Goroka and Lutheran School of Nursing.	No
Republic of Marshall Islands	54–46	No	Diploma: College of the Marshall Islands Nursing School	No
Samoa	27–25	No	Bachelor of nursing, followed by post-graduate midwifery training: National University of Samoa or University of the South Pacific	No
Solomon Islands	71–37	No	Diploma, Solomon Islands School of Higher Education	No
Timor Leste	56	No	Yes	No
Tokelau		No	Diploma: Fiji School of Nursing	No
Tonga	23–22	No	Diploma: Queen Salote School of Nursing, Tonga	No
Tuvalu	37–36	No	Diploma: Fiji School of Nursing	No
Vanuatu	34–30	No	Vanuatu College of Nursing Education	Vanuatu College of Nursing Education

UNFPA – United Nations Population Fund, NZAID – New Zealand Aid

## BARRIERS TO THE DEVELOPMENT OF LEADERS

Leadership positions within the public sector, whether in academia or public health, are often not well remunerated, and there is therefore much less financial incentive as compared to private or overseas practice (38,39). The hierarchy and bureaucracy within health departments and across other government departments may make it difficult for leaders to have influence over the broad and multi-sectoral activities outlined within maternal and child health plans. Countries in which there are opportunities for training and accreditation in overseas institutions are particularly vulnerable to attrition of the most highly trained individuals either to migration or the private sector (38-40). Individuals at the district level have much lower opportunities for further training (41). In addition to these are personal barriers (eg, time constraints and family commitments), political barriers (tolerance of organised professional associations), and cultural barriers (influence of age, gender and ethnicity on being able to take on national leadership positions).

In many countries, leading the translation of policy into practice is met by key challenges including complex national bureaucratic health systems, inadequate links between government departments (such as maternal and child health departments, finance and pharmaceuticals departments), and lack of human resources and technical capacity in peripheral areas.

## APPROACHES TO IMPROVING LEADERSHIP THROUGH ADVANCED TRAINING

Increasing the capacity of nurses and doctors through local post-graduate training would encourage retention of these leaders within the public sector (42). There have been several models for this in the Region (**Table 2**), including paediatric training for doctors, child health nurses, and nurse practitioners.

In Fiji and Vanuatu, nurse practitioners – mid-level health workers (usually graduate nurses) – are trained to function independently in remote settings, across several disciplines not specifically in child health (26,29). Evaluation of this program in Fiji showed high acceptance by candidates and the community, although further paediatric training was identified as a need if nurses are to practice independently in settings with no medical officer. Fiji and PNG currently train post-graduate nurses from surrounding countries, but this is limited by cost of international travel and accommodation, and coverage across the Pacific is very low.

In the Pacific Region post-graduate training for doctors in child health occurs in only PNG and Fiji. In PNG more than 40 paediatricians have been trained in the last 20 years, and there has been a doubling of capacity for provincial-level leadership in the last decade.

Another example from a Group 1 priority country in the Western Pacific is Lao PDR. In this country a Paediatric Residency Program – a 3-year course that trains doctors from each province in the central tertiary hospital in clinical paediatrics and public child health – has been running for over 12 years. A survey of graduates showed that Lao paediatricians have leadership roles in provincial hospitals where they teach and supervise other staff, and the Paediatricians Network is increasingly leading child health programs (30).

## DISCUSSION

There is indirect evidence to guide approaches for strengthening leadership in the Pacific and poor Asian countries. A number of approaches seem appropriate.

### Greater support for professional associations and training

Effective leadership at a higher political and policy level can be transformational for countries’ health outcomes (12,13). However, there are other important facets of leadership for child health that also warrant more attention than has recently been given.

Effective paediatric, obstetric and midwifery associations have been important historically in the development of maternal and child health services. They require a critical mass of committed and well-trained professionals, working closely with the ministry of health. The challenge in many Pacific Island countries and in East Timor is the insufficient numbers of such people, and a lack of cohesive coordination. At a Pacific regional level the scale-up of post-graduate paediatrician training should be a medium term goal, and is a necessary pre-requisite to having this critical mass. In the immediate term, there are individuals in each country who fulfil leadership roles, and these need to be properly recognised and supported by ministries of health and development partners. The involvement of such people in policy decisions, particularly clinical leaders in provinces and districts, will be particularly important for bridging the policy-implementation gap.

There is a need for post-graduate training for nurses in child health, and these contribute to the critical mass. Nurse training needs to be clinically orientated and practical, but also equip nurses with basic public health and practical epidemiological skills necessary to support national child health programs and understand health information systems. Many countries in the Pacific would be able to deliver this training locally, therefore saving costs and avoiding the problems of overseas migration of regional and international training. The PNG diploma of child health or the Fiji Nurse Practitioner training with additional child health components may provide the comprehensive clinical and public health training model that is needed in the Pacific context.

Gradually, countries need a larger group of public child health professionals skilled in a variety of areas. This is being achieved in PNG through their now well-established post-graduate paediatric training program (22). In Lao PDR graduate paediatric doctors work in almost all provinces and maintain a collegiate professional network, increasingly engaged in policy work, standard setting and research. The approach in each country will differ, but there are common principles shared by the PNG and Lao programs that can be used in other countries (**Table 3**).

**Table 3:** Features of post-graduate paediatric education that can promote leadership

Aim to train independent child health nurse practitioners, skilled in clinical diagnosis, basic treatment and procedures and when to refer
Align the course content with the national child health plan, clinical guidelines and public child health programs
Teach an understanding of global approaches that can be adapted nationally
Convey an understanding of locally important burdens of disease and mortality
Learn about national and local systems of surveillance and data
Introduce training in quality of care, and minimal standards
Ensure that intake policies promote geographical and ethnic representation that supports equity, ie, from rural and remote provinces where human resources need most strengthening
Encourage input into the course content and structure from the national paediatric association
Follow-up and ongoing mentorship: provide mechanisms for graduates working at the provincial and district level to communicate and obtain advice from training institutions, senior clinicians, or academics
Establish a child health professional organization, including paediatricians and child health nurses aligned to national priorities. It is time to put effort and resources into CPD that is led by local institutions and reflects national priorities. CPD does not have to be expensive in developing countries and can be introduced at scale, led by the national paediatric association (18)
Involvement in local research, especially that which contributes directly to critical issues in child health policy or strengthening national data systems builds capacity for leadership. There are challenges to achieving this in countries with limited manpower, but positive examples from PNG and Laos.

To keep up with the changing content and landscape of child health, there is a need for continuing professional development (CPD) for both nurses and doctors. This creates challenges for training institutions, professional bodies and ministries of health. The most common forms of CPD in the Pacific in recent decades have been multiple in-service training programs supported by donor partners to implement vertical programs. These continue to be driven by availability of funds and trends in global health, but have not been part of coordinated programs that reflect and are aligned to national priorities. It is time to put effort and resources into CPD that is led by local institutions and reflects national priorities. CPD does not have to be expensive in developing countries and can be introduced at scale, led by the national paediatric association (18).

Involvement in local research, especially that which contributes directly to critical issues in child health policy or strengthening national data systems builds capacity for leadership. There are challenges to achieving this in countries with limited manpower, but positive examples from PNG and Laos.

### Common goals

Adaptation and implementation of the Western Pacific Regional Child Survival Strategy can be used as a starting point to work towards unified goals, and to provide existing professional organisations for maternal and child health with a framework for supporting the implementation of local plans. Uptake of this Strategy by smaller Pacific Island countries has been poor (33).

The national implementation of the Integrated Management of Childhood Illness (IMCI) at the community and primary health facility level and the WHO Pocketbook of Hospital Care for Children at the hospital level are also unifying goals that address issues of health service quality and the new technical content of child health (43). There is a need to provide sustained support to these initiatives in a coordinated way, by countries, donor partners and agencies, and to evaluate them.

Development and implementation of a National Child Health Plan, which brings together the strategies in the Child Survival Strategy, interventions to improve quality of care, other vertical programs as they relate to children (TB, HIV, malaria), immunization, newer areas of focus (such as child disability, child protection, neonatal care, adolescent health, sub-specialty paediatrics) and human resource plans, can provide the framework for a comprehensive child health service, and requires many skilled leaders (11).

Providing talented people in provincial areas with national portfolio responsibility for areas of child health can keep them in touch with national progress, assist in engagement and job satisfaction, and build enthusiasm for working towards shared national goals.

### Providing networks of support, and opportunities for dialogue, contribution and research

Mentorship for individuals working in remote areas is important, and rarely done in the Pacific and rural Asia. Successful examples of this include the Thailand Rural Doctor Coaching Program (14), and less formal approaches being taken in Laos and PNG. There are increasing opportunities to build support networks with modern methods of communication and social networking.

Annual meetings where child health professionals convene to discuss policy and clinical issues have been running for many years in PNG and Laos, and are an essential element of supporting individuals in remote areas. Dialogue in such meetings between provincial paediatricians and provincial public health administrators has been important in improving the technical and policy understanding between professional groups. The sharing of local research and the interpretation of global research in the local context has also been important.

There are currently regional associations for child health, such as the Asian Pacific Paediatric Association and the Pasifika Medical Association, and these have the potential to provide a forum for sharing of ideas and experiences of senior leaders from each Pacific Island country.

## CONCLUSIONS

Strong local leadership is needed to address the complexities and challenges of child health, at national, provincial and district level, among paediatricians, child health nurses and policy makers. Each country’s efforts at this will be different, but it is a need not currently met in a serious way by governments or other agencies. It requires collaboration between governments, professional associations, training institutions, and decentralized health authorities. A substantial increase in skilled human resources in the Pacific Region is necessary if child health services are to be fully developed and health targets are to be reached. This will take a reconsideration of the complex roles that need to be played by clinical and public health staff and their professional development needs. The recent interest in strengthening health systems needs to include support for local institutions of training – large and small – and support for the roles of professional associations of paediatrics.
